# Recent Advances in Nonprecious Metal Oxide Electrocatalysts and Photocatalysts for N_2_ Reduction Reaction under Ambient Condition

**DOI:** 10.1002/smsc.202000069

**Published:** 2021-03-27

**Authors:** Tong Xu, Jie Liang, Shaoxiong Li, Zhaoquan Xu, Luchao Yue, Tingshuai Li, Yonglan Luo, Qian Liu, Xifeng Shi, Abdullah M. Asiri, Chun Yang, Xuping Sun

**Affiliations:** ^1^ Institute of Fundamental and Frontier Sciences University of Electronic Science and Technology of China Chengdu Sichuan 610054 China; ^2^ College of Chemistry and Materials Science Sichuan Normal University Chengdu Sichuan 610068 China; ^3^ College of Chemistry Chemical Engineering and Materials Science Shandong Normal University Jinan Shandong 250014 China; ^4^ Chemistry Department Faculty of Science & Center of Excellence for Advanced Materials Research King Abdulaziz University P.O. Box 80203 Jeddah 21589 Saudi Arabia

**Keywords:** ambient conditions, electrocatalysis, N_2_ reduction reaction, nonprecious metal oxides, photocatalysis

## Abstract

NH_3_ plays an indispensable role in agriculture, fertilizer production as well as in the chemical industry. However, its large‐scale production still relies deeply on the century‐old Haber–Bosch process under high temperature and pressure along with greenhouse gas emission and fossil fuel consumption. The electrocatalytic and photocatalytic N_2_ reduction reactions (NRRs) for NH_3_ production are favorable approaches to avoid these issues because they are carbon‐neutral and energy‐saving. Recently, the nonprecious metal oxides (NPMO) have gathered the most attention due to their ease of synthesis, controllable stability, lower cost, and environmental friendliness. Herein, the recent advances in NPMO electrocatalysts and photocatalysts for the NRR are narrated and the strategies to improve the poor NRR activity of pristine NPMOs by heteroatom doping to engineer their surface‐active sites and introduction of oxygen vacancies are highlighted. A brief summary of and the future perspective on this research field are also presented.

## Graphical abstract

The recent progress in designing and developing nonprecious metal oxides for electrocatalytic and photocatalytic N_2_ reduction is summarized. Strategies to boost the catalytic performance are discussed, which include introducing oxygen vacancies, heteroatom doping, and hybridization with carbon. A brief summary of and the future perspective on this research field are also proposed.

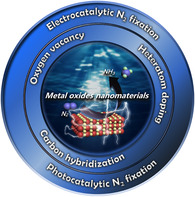

## 1 Introduction

**Figure 1:**
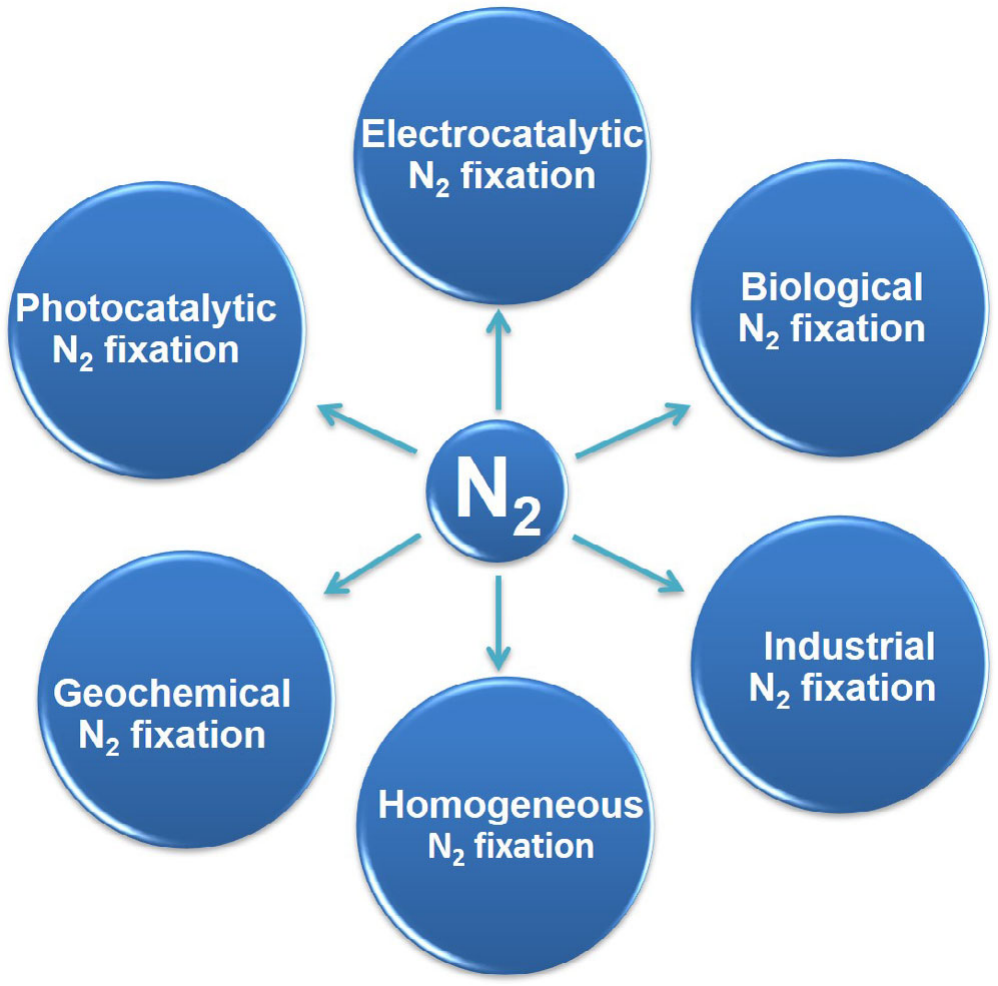
Schematic diagram of different reaction pathways for N_2_ fixation.

NH_3_, which plays an indispensable role in agriculture, fertilizer production as well as the chemical industry^[^ ^1^, ^2^ ^]^ is one of the most fundamental constituents of the modern industrial society. More importantly, NH_3_ is a carbon‐free energy carrier with no CO_2_ emission at final decomposition. In consideration of its high energy density and eco‐friendly combustion products, NH_3_ is promising as a feasible substitute for H_2_ for the modern energy economy. Although N_2_ accounts for more than 78% of the atmosphere, the usage of N_2_ is very painstaking because of the strong triple bond.^[^ ^3^ ^]^ At present, there are mainly three ways of N_2_ fixation (**Figure** **1**): 1) Industrial‐scale fixation (N_2_ + 3H_2_ → 2NH_3_) is the century‐old Haber–Bosch process under high temperature and pressure along with greenhouse gas emission and fossil fuel consumption;^[^ ^4^ ^]^ 2) biologically, NH_3_ synthesis is under the excitation of nitrogenase enzymes in the perseverance of H_2_O, electrons, and atmospheric N_2_ under mild conditions;^[^ ^5^, ^6^ ^]^ 3) geochemically, it is a high‐energy fixation process, such as lightning. However, the NH_3_ yields of biological and geochemical ways are subtle and unstable. Large‐scale NH_3_ synthesis still relies on the energy‐intensive Haber–Bosch process. Therefore, it is of great value to develop alternative methods for NH_3_ synthesis.

In recent years, there has been ample research on electrocatalytic and photocatalytic reactions, especially the CO_2_ reduction reaction (CRR),^[^ ^7^, ^8^, ^9^ ^]^ O_2_ evolution reaction (OER),^[^ ^10^, ^11^ ^]^ O_2_ reduction reaction (ORR),^[^ ^12^, ^13^ ^]^ and H_2_ evolution reaction (HER).^[^ ^14^, ^15^ ^]^ Electrocatalysis and photocatalysis enable the N_2_ reduction reaction (NRR) to make NH_3_.^[^ ^16^, ^17^ ^]^ Compared with the conventional Haber–Bosch process, the electrocatalytic and photocatalytic process under ambient conditions reduces the energy input, simplifies the reaction steps, and eases the pressure on the environment.^[^ ^18^, ^19^ ^]^ But the process still needs efficient catalysts to break the strong N≡N. Therefore, designing excellent catalysts to boost the electrocatalytic and photocatalytic NRR efficiencies is highly demanded.

Encouragingly, nonprecious metal oxides (NPMOs) have been established as promising materials for a wide variety of applications (such as supercapacitors, sensors, and in the biomedical fields) because of their ease of synthesis, chemical stability, and low cost.^[^ ^20^, ^21^, ^22^ ^]^ In addition, transition metals in NPMOs show paramagnetic activity and variable oxidation states. Because of these merits, NPMOs have been investigated extensively as NRR catalysts recently. Some reviews on the NRR have been documented,^[^ ^23^, ^24^, ^25^ ^]^ which provide in detail the recent progress on the design of NRR electrocatalysts and photocatalysts. However, few of them focused on NPMOs. Accordingly, a recent review on NPMOs toward the NRR for electrocatalysis and photocatalysis is required. In our review, we summarize the recent progress of NPMO catalysts toward ambient NRR. We highlight the tactics to boost the scanty NRR activity of pristine NPMOs via heteroatom doping to engineer their surface‐active sites and introducing oxygen vacancies (OVs). Eventually, we also propose a brief summary of and future perspective on this research field.

## 2 Basic Understanding of the NRR

### 2.1 Electrocatalytic N_2_ Reduction

Electrocatalytic N_2_ fixation is regarded as an auspicious and green means to achieve mild N_2_‐to‐NH_3_ conversion with adsorption, activation, and desorption processes.^[^ ^26^, ^27^, ^28^, ^29^ ^]^ The electrocatalytic NRR process encompasses the following elementary steps: 1) diffusion of N_2_ into the surface; 2) activation of N_2_ molecules to intermediates via the participation of protons and multiple electron transfer; and 3) desorption of the reduced product from the surface. In the concrete process, the water oxidation and N_2_ reduction occur respectively in the anode and cathode three‐electrode system. The corresponding reactions are shown as follows.

Acidic condition

(1) $$\text{anode}:3H_{2}O\to 3/2O_{2}+9H^{+}+6e^{-}$$

(2) $$\text{cathode}:6H^{+}+6e^{-}+N_{2}\to 2{\text{NH}}_{3}$$

Alkaline condition

(3) $$\text{anode}:6{\text{OH}}^{-}\to 3/2O_{2}+3H_{2}O+6e^{-}$$

(4) $$\text{cathode}:6H_{2}O+N_{2}+6e^{-}\to 2{\text{NH}}_{3}+6{\text{OH}}^{-}$$

(5) $$\text{overall reaction}:{\text{ N}}_{2}+3H_{2}O\to 3/2O_{2}+2{\text{NH}}_{3}$$

Compared with the Haber–Bosch process, electrocatalytic N_2_ fixation is mild because it utilizes electrical energy instead of thermal energy. In addition, water is the hydrogen source, avoiding the use of fossil fuels. To date, electrochemical NH_3_ synthesis has been demonstrated on precious metals, including Ru,^[^ ^30^ ^]^ Au,^[^ ^31^, ^32^ ^]^ Pd,^[^ ^33^, ^34^ ^]^ and Ag.^[^ ^35^ ^]^ Although the catalytic performance of noble metal catalysts is good, the high cost and scarcity impose great confinements on large‐scale production. Therefore, it is urgent to find more NPMO catalysts for the NRR.

### 2.2 Photocatalytic N_2_ Reduction

The photocatalytic method is deemed to be environmentally friendly and energy‐saving for NH_3_ production as this process operates mildly and takes advantage of sustainable solar energy. Different from electrocatalysis, the photosynthesis of NH_3_ directly produces NH_3_ from sunlight, N_2_, and H_2_O. Since Schrauzer and Guth first published their study on the photoreduction of N_2_ in 1977,^[^ ^36^ ^]^ scientific endeavors on finding effective photocatalysts for N_2_ reduction have increased exponentially.^[^ ^37^, ^38^ ^]^ Nevertheless, the conversion efficiency is still low due to the recombination of electron–hole pairs and limited active sites.

In general, N_2_ photofixation involves the following critical steps: At first, the semiconductor's conduction band and valence band generate photoinduced holes and electrons to form hole/electron pairs (h^+^/e^−^) after the adsorption of UV light or visible light. Charge separation and photoinduced carrier migration are the second step of N_2_ photofixation. Finally, e^−^ diffuse across the photocatalyst's surface and reach the active sites, on which the absorption of N_2_ molecules and the following conversion processes take place. Meantime, sacrificial agents, such as H_2_O, are oxidized by h^+^. The specific reactions are shown as follows

(6) $$2H_{2}O+4h^{+}\to 4H^{+}+O_{2}$$

(7) $$6H^{+}+N_{2}+6e^{-}\to 2{\text{NH}}_{3}$$

(8) $$\text{overall}:2N_{2}+6H_{2}O\to 3O_{2}+4{\text{NH}}_{3}$$

As recombination of e^−^ and h^+^ and the limited active sites of the photocatalysts hinder the photocatalytic efficiency, developing photocatalysts with enriched active sites and small bandgaps (more generation of h^+^/e^−^) and improving the utilization of charge carriers simultaneously appear to be particularly important for the photosynthesis of NH_3_.

### 2.3 Theoretical Studies of N_2_ Reduction

The difficulty in activating N_2_ lies in the following aspects. 1) N_2_ as a Lewis base shows low proton affinity, nonpolarity, and negatron affinity. 2) The high‐energy N≡N bond (941 kJ mol^−1^) renders the N_2_ molecule inert and stable. 3) Also, the energy differences between the lowest vacant molecular orbital (LUMO) and the highest vacant molecular orbital (HOMO) in N_2_ do not promote electron transfer.^[^ ^23^ ^]^ Thus, it is difficult to activate N_2_.

**Figure 2:**
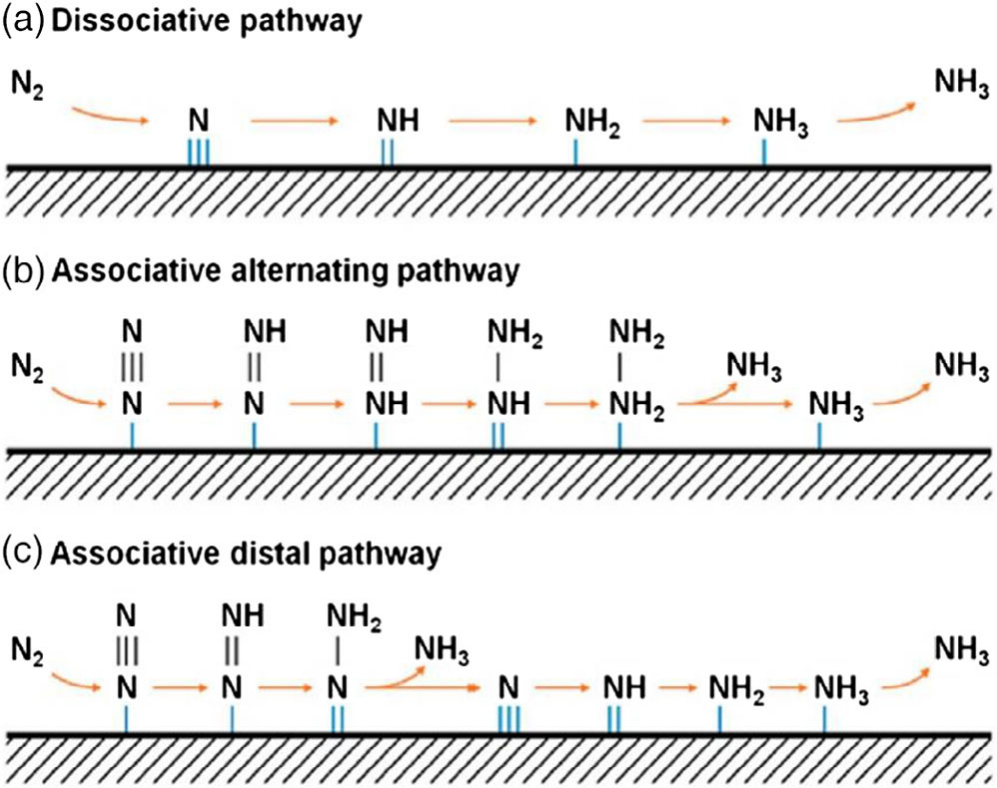
The general reaction mechanisms of N_2_ fixation. a) Dissociative pathway. b) Associative alternating pathway. c) Associative distal pathway. Reproduced with permission.^[^ ^23^ ^]^ Copyright 2020, American Chemical Society.

Tafel‐type reactions are defined due to H atoms on the electrode surface combination with adsorbed N_2_H_*x*_ or NH_*x*_ species, and the combination of adsorbed N_2_H_*x*_ or NH_*x*_ species with protons and electrons is called a Heyrovsky‐type reaction. Heyrovsky‐type reactions include both associative and dissociative mechanisms (**Figure** **2**). In the process, N_2_ molecules are first absorbed on the surface of the catalyst and further the N≡N is cleaved. And NH_3_ releases with the final N—N bond cleavage, which mainly concerns with Haber–Bosch process. The biological nitrogenase process is related to the associative pathway. The associative mechanism can be subdivided into an alternate pathway and a distal pathway. In the alternating process, the two N atoms are successively hydrogenated till the final N—N bond is broken and NH_3_ is released. The N atoms far from the catalyst surface are first hydrogenated and released as NH_3_, which leaves an absorbed N atom on the surface of the catalyst and further hydrogenates to synthesize NH_3_ in the distal pathway.^[^ ^23^ ^]^

### 2.4 Selectivity for N_2_ Reduction

**Figure 3:**
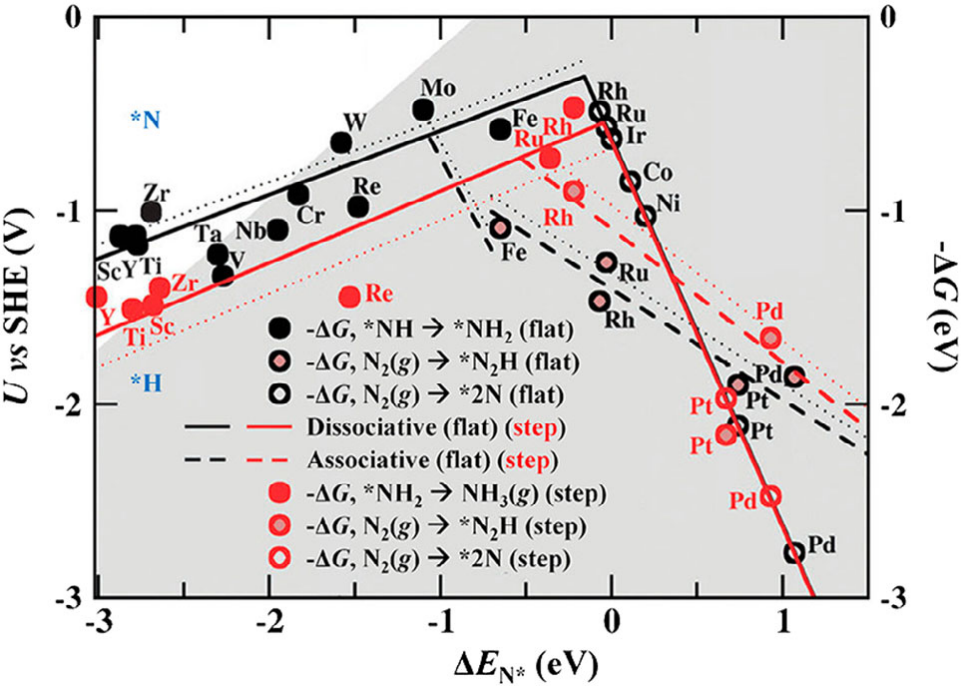
Volcano diagrams obtained by plotting the theoretical limiting potential for N_2_ reduction via a Heyrovsky‐type mechanism against the chemisorption energy of N adatoms on a range of flat (black) and stepped (red) transition‐metal surfaces. Reproduced with permission.^[^ ^23^ ^]^ Copyright 2020, American Chemical Society.

In the process of electrocatalytic N_2_ reduction, the breaking of the strong N≡N requires a reduction potential where the HER readily occurs, leading to a low efficiency and selectivity for NH_3_ production. Therefore, current progress in the electrochemical NRR is inhibited by the intense competition from the HER due to the affinity of the H atom with metals. It is imperative to overcome the selectivity challenge to suppress water electrolysis and enhance solid catalyst–gas interactions simultaneously. Slowing down proton transformation to the electrocatalyst surface is the main principle to improve the selectivity. Rational design of catalysts by computational methods makes it possible to prepare new materials that outperform state‐of‐the‐art commercial catalysts.^[^ ^39^ ^]^ Although there are rare specific theoretical reports on photocatalytic N_2_ reduction, it is universal in electrocatalytic N_2_ fixation using density functional theory (DFT) calculations to explain concert reaction pathways and mechanisms.^[^ ^40^ ^]^ As shown in **Figure** **3**, a volcano‐shaped plot is obtained by DFT calculations that shows whether the metal surface is overlapped with adsorbed N atoms or H atoms. Rh, Ru, Mo, and Fe are likely to show the highest catalytic activity for N_2_ reduction because these metals locate on top of the volcanoes. Nevertheless, these metals’ surfaces are covered with H atoms and the HER will follow when they get negative potentials. The transition metals on the right side of the volcano plots need more negative potentials to conduct the NRR. However, with the negative potential increasing, H atoms are absorbed in these surfaces and the HER becomes the dominant reaction.^[^ ^23^ ^]^ According to calculations and Figure 3, some transition metals on the left side of the volcano plots probably bind N adatoms more strongly than H adatoms at −1 to −1.5 V versus a standard hydrogen electrode (SHE). Therefore, it gives us suggestions to rationally design NRR catalysts based on these metals with higher selectivity and Faradaic efficiencies (FEs).

To suppress the HER, some external proposals are suggested. 1) Adding alkali metal ions such as Li^+^, Na^+^, and K^+^ into the electrolyte to restrict the H_2_ evolution.^[^ ^41^ ^]^ Hao et al. reported that K^+^ can suppress the HER on bismuth catalysts in aqueous solution due to the prevention of proton migration from the bulk solution to the electrode surface. The FE increased to 66%.^[^ ^42^ ^]^ 2) Designing a hydrophobic layer to inhibit the access of water molecules to the encapsulated active surfaces. Ling and co‐workers used the zeolitic imidazolate framework (ZIF)‐71 with superhydrophobicity to cover a catalyst surface and used the metal–organic frameworks (MOFs) with high gas sorptivity to absorb N_2_.^[^ ^43^ ^]^ Zhang and co‐workers, inspired by nitrogenases, utilized thiols to form self‐assembled monolayers (SAMs) on the surface of Ru. The organic and hydrophobic nature of the SAM limits proton transfer while allowing N_2_ transport to the surface of the catalyst.^[^ ^44^ ^]^ Inspired by the hydrophobic hairs of aquatic arachnids, Wakerley et al. developed an analogous multiscale hydrophobic surface by modifying dendritic Cu with a monolayer of waxy alkanethiol.^[^ ^45^ ^]^ 3) Changing the pH value to improve the FE. The HER can be weakened by decreasing the H^+^ concentration in a weakly acidic electrolyte.^[^ ^41^ ^]^

## 3 Recent Advances in NPMO Electrocatalysts for the NRR

In recent years, an increasing number of researchers have been paying attention to resource‐rich elements in the earth. In this section, we sum up the advances in NPMO electrocatalysts based on groups of the period table for the NRR because NPMOs of the same group have similarities.

### 3.1 Pristine NPMOs

Sc, Y, and La in third subgroup have NRR activity. Our group reported that Y_2_O_3_ nanosheets and La_2_O_3_ nanoplates were active for the NRR.^[^ ^46^, ^47^ ^]^ Later, Sc and Y as single‐atom NRR electrocatalysts with high FEs of 11.2% and 12.1% were investigated.^[^ ^48^ ^]^ Although they are rare‐earth elements, it is worth taking more efforts to study their catalytic properties toward the NRR because of the following reasons: 1) The abundant electron orbitals are conducive to N_2_ activation and 2) their stability is great.

In the fourth subgroup, Ti compounds are stable and low‐cost. Pristine TiO_2_ (p‐TiO_2_) achieved an NH_3_ yield of 0.17 × 10^−10^ mol s^−1^ cm^−2^ and an FE of 0.95%.^[^ ^49^ ^]^ With the toughness, hardness, and wear resistance properties, more research has been concentrated on ZrO_2_. In our group, Xu et al. reported ZrO_2_ nanoparticles as efficient electrocatalysts toward the NRR, presenting an NH_3_ yield of 24.74 μg h^−1^ ${\text{mg}}_{\text{cat}\text{.}}^{-1}$, with an FE of 5.0% in 0.1 m HCl.^[^ ^50^ ^]^ When tested in 0.1 m KOH and 0.2 m phosphate buffer solution (PBS), the FEs are lower than that in 0.1 m HCl. This is because the high H^+^ concentration limits the HER. Over recent years, there has been ample research on TiO_2_ in the NRR. However, reports about ZrO_2_ in the NRR are limited. Zr‐based materials need more dedication to further widen their applications in the NRR.

**Figure 4:**
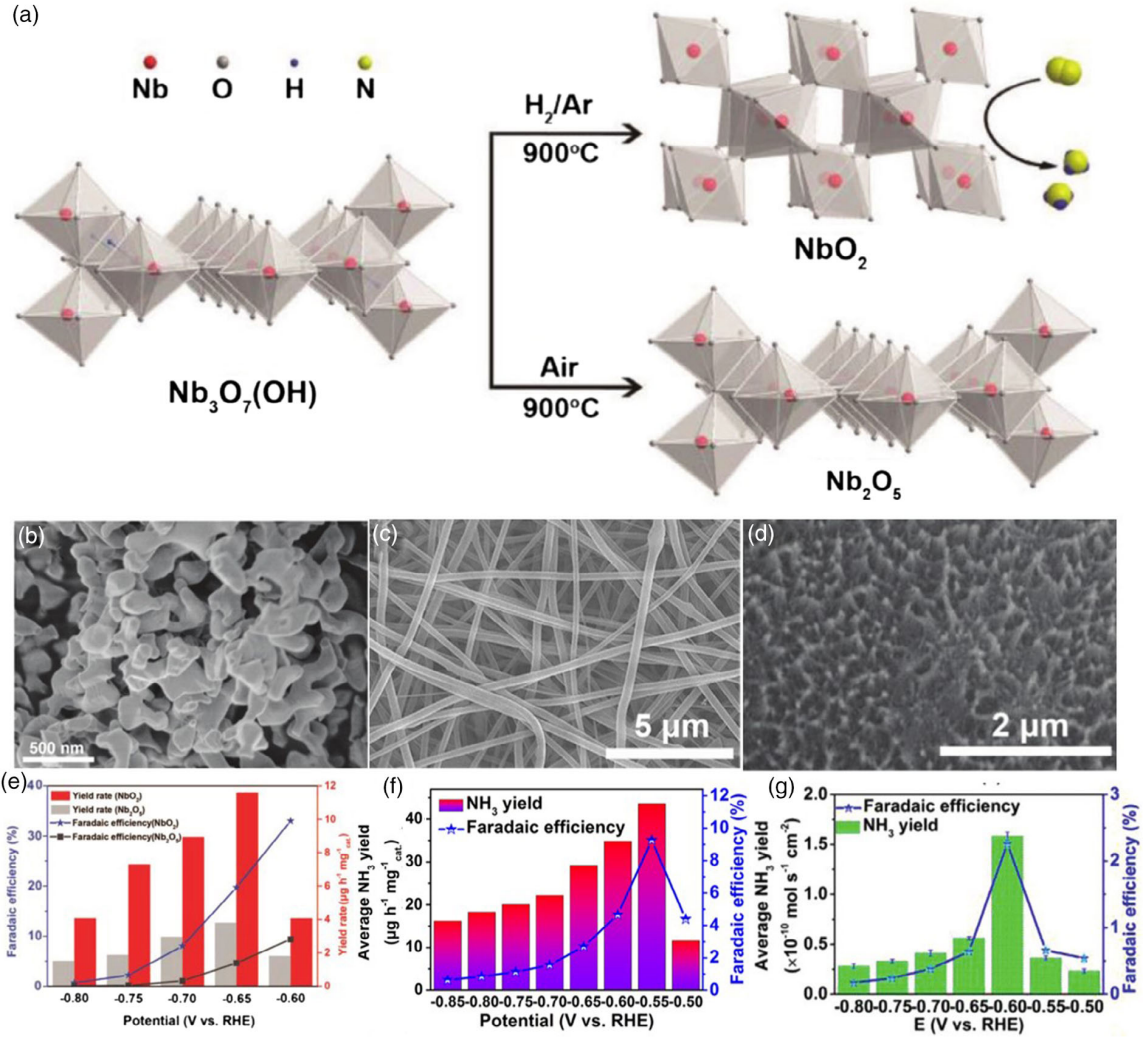
a) Schematic for the synthesis procedure of NbO_2_ and Nb_2_O_5_. SEM images of b) NbO_2_, c) Nb_2_O_5_ nanofibers, and d) Nb_2_O_5_ nanowire array. Average NH_3_ yields and FEs for e) NbO_2_, f) Nb_2_O_5_ nanofibers, and g) Nb_2_O_5_ nanowire array at each given potential. a,b,e) Reproduced with permission.^[^ ^51^ ^]^ Copyright 2018, Wiley‐VCH. c,f) Reproduced with permission.^[^ ^52^ ^]^ Copyright 2018, Elsevier. d,g) Reproduced with permission.^[^ ^53^ ^]^ Copyright 2019, Royal Society of Chemistry.

In the fifth subgroup, Nb compounds are significant in heterogeneous catalysis. NbO_2_ is near the top of the volcano and DFT calculations reveal that NbO_2_ with the (110) facet is an excellent NRR electrocatalyst due to the high capability for N_2_ reduction to NH_3_ while suppressing the competing water reduction. NbO_2_ and Nb_2_O_5_ were recently developed by Huang et al. to catalyze the NRR, and a schematic illustration of their synthesis procedure is shown in **Figure** **4**a.^[^ ^51^ ^]^ In their research, they prepared both NbO_2_ (Figure 4b) and Nb_2_O_5_ from a Nb_3_O_7_(OH) nanorod precursor and then annealed in H_2_/Ar and air atmosphere, respectively. After the NRR test, NbO_2_ performed brilliantly on average NH_3_ yields and FE compared to Nb_2_O_5_ (Figure 4e). It can be seen that the oxidation state of Nb can result in a different electrocatalytic NRR activity. The excellent NRR performance of NbO_2_ is attributed to the partly occupied *d* orbitals of ${\text{Nb}}_{4}^{+}$ that can form π backbonding with N_2_. After six recycling tests and 12 h long electrolysis in 0.05 m H_2_SO_4_, NbO_2_ was still stable. Another kind of metal oxide of Nb has been reported. For example, Han et al. prepared crystalline Nb_2_O_5_ nanofibers (Figure 4c) by electrostatic spinning followed by air annealing and such an electrocatalyst achieved an NH_3_ yield of 43.6 μg h^−1^ ${\text{mg}}_{\text{cat}\text{.}}^{-1}$ and an FE of 9.26% (Figure 4f).^[^ ^52^ ^]^ In addition, Kong et al. also reported a Nb_2_O_5_ nanowire array (Figure 4d) on carbon cloth, showing an NH_3_ yield of 1.58 × 10^−10^ mol s^−1^ cm^−2^ and an FE of 2.26% (Figure 4g).^[^ ^53^ ^]^ The NRR performance of the same oxides with different morphologies has slight differences. Based on this, when designing the catalyst, we can acquire the best performance by adjusting the morphology. Zhu and co‐workers reported a novel flower‐like porous Ce_1/3_NbO_3_ perovskite as an NRR electrocatalyst due to the synergistic effect of the Ce atom.^[^ ^54^ ^]^

In the same subgroup, V has a vital role in the nitrogen enzyme to catalyze N_2_ fixation in biology under ambient conditions.^[^ ^55^ ^]^ V oxide nanomaterials have been used in the field of batteries and electrolysis. In the NRR field, hollow microsphere VO_2_ performed efficaciously and steadily with an NH_3_ yield of 14.85 μg h^−1^ ${\text{mg}}_{\text{cat}\text{.}}^{-1}$ and FE of 3.97%.^[^ ^56^ ^]^

**Figure 5:**
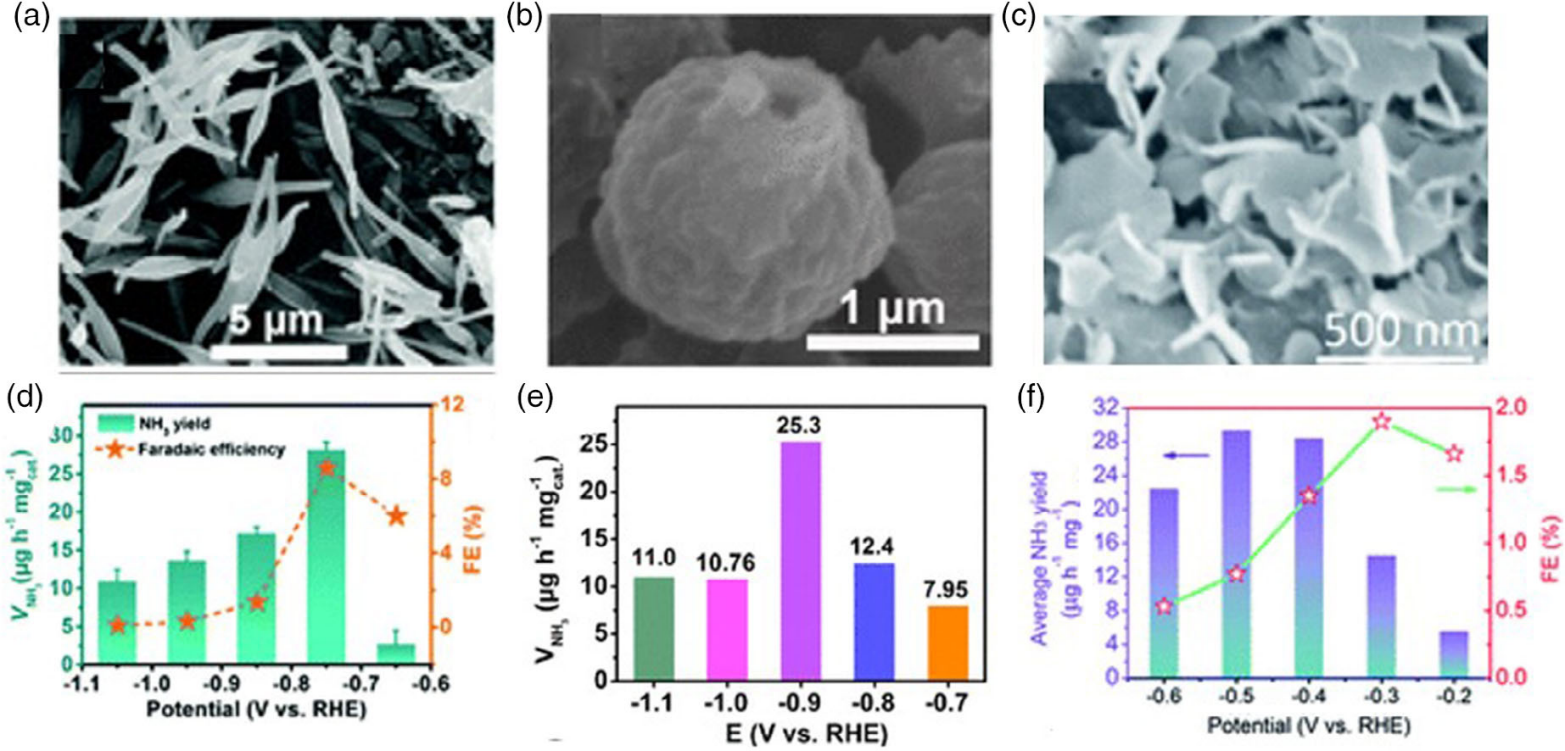
SEM images of a) Cr_2_O_3_ nanofibers, b) hollow COMs, and c) MoO_3_ nanosheets. NH_3_ yields and FEs of d) Cr_2_O_3_ nanofibers, e) hollow COMs, and f) MoO_3_ nanosheets at different potentials. a,d) Reproduced with permission.^[^ ^57^ ^]^ Copyright 2018, Royal Society of Chemistry. b) Reproduced with permission.^[^ ^58^ ^]^ Copyright 2018, American Chemical Society. c,e,f) Reproduced with permission.^[^ ^62^ ^]^ Copyright 2017, Royal Society of Chemistry.

In the sixth subgroup, Cr and Mo have attracted much attention. Du et al. synthesized Cr_2_O_3_ nanofibers by electrospinning of polyacrylonitrile/chromium acetate by air annealing.^[^ ^57^ ^]^ **Figure** **5**a shows a concrete image of the Cr_2_O_3_ nanofibers after annealing, the NH_3_ yield and FE achieved by which are 28.13 μg h^−1^ ${\text{mg}}_{\text{cat}\text{.}}^{-1}$ and 8.56%, respectively (Figure 5d). In addition, Zhang et al. reported multishelled hollow Cr_2_O_3_ microspheres (MHCOMs) (Figure 5b) serving as a catalyst for the NRR.^[^ ^58^ ^]^ For comparison, Cr_2_O_3_ microspheres (COMs) and Cr_2_O_3_ nanoparticles (CONPs) were also prepared in Zhang's work. Of note, the NRR activity of the multishelled hollow Cr_2_O_3_ (Figure 5e) was higher than those of COMs and CONPs. This is because the adopted N_2_ in the hollow inner surface of the microspheres has higher frequency collisions and the concentration of the species in the rate‐determining step increased due to the confined effect of the cage. This further demonstrates that we can get the best performance of catalysts by morphology modulation.

Mo is a significant element in nitrogen enzyme that catalyzes N_2_ fixation in biological fields.^[^ ^59^, ^60^ ^]^ Recently, Yang et al. reported that in NRR electrocatalysis, Mo nanofilms attached an NH_3_ yield of 3.09 × 10^−10^ mol s^−1^ cm^−2^ and an FE of 0.72%.^[^ ^61^ ^]^ Han et al. prepared MoO_3_ by a one‐step hydrothermal strategy. Figure 5c shows the specific SEM image. Figure 5f shows the NRR property, with the optimal NH_3_ yield and FE attained being 29.43 μg h^−1^ ${\text{mg}}_{\text{cat}\text{.}}^{-1}$ and 1.90%, respectively.^[^ ^62^ ^]^

In the seventh subgroup, the Mn element is demonstrated to tremendously boost the catalytic performance of nitrogenases by extraction from the photosynthetic bacterium.^[^ ^63^, ^64^ ^]^ As an earth‐abundant metal, Mn‐based oxides are active for the NRR. Wu et al. reported that Mn_3_O_4_ nanocubes, synthesized by hydrothermal reaction at 120 ° for 12 h, attained an NH_3_ yield of 11.6 μg h^−1^ ${\text{mg}}_{\text{cat}\text{.}}^{-1}$ and an FE of 3.0%.^[^ ^65^ ^]^ Furthermore, Wang et al. demonstrated that MnO particles on Ti mesh (MnO/TM) were also an active NRR catalyst with an NH_3_ yield of 1.11 × 10^−10^ mol s^−1^ cm^−2^ and FE of 8.02%.^[^ ^66^ ^]^ The oxidation state of Mn can result in different electrocatalytic NRR activity.

In the eighth group, Fe is one of the most abundant non‐noble metals. Moreover, Fe as a natural nitrogenase is capable of catalyzing N_2_ fixation in ambient atmosphere. Liu et al. and Xiang et al. reported spinel Fe_3_O_4_ nanorods on a Ti mesh (NH_3_ yield : 5.66 × 10^−11^ mol s^−1^ cm^−2^, FE: 2.6%)^[^ ^67^ ^]^ and Fe_2_O_3_ nanorods (NH_3_ yield: 15.9 μg h^−1^ ${\text{mg}}_{\text{cat}\text{.}}^{-1}$, FE: 0.94%)^[^ ^68^ ^]^ as NRR electrocatalysts, respectively. Compared with Fe_2_O_3_ nanorods, the spinel‐type‐structure Fe_3_O_4_ nanorods have higher conductivity, which contributes to enhancement of the electrocatalytic NRR performance.

**Figure 6:**
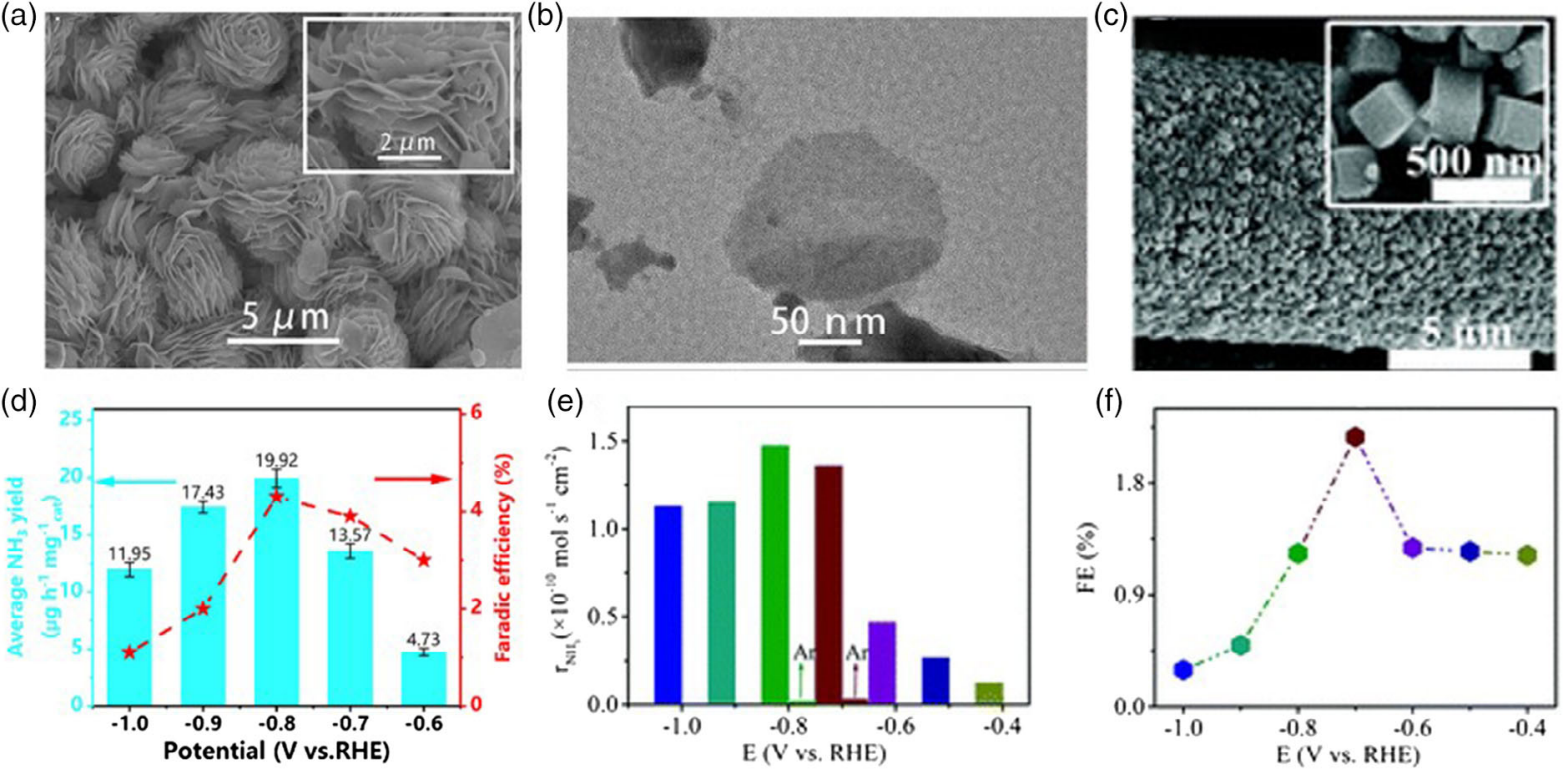
a) SEM image and b) TEM image of flower‐like β‐Bi_2_O_3_. c) SEM images of cubic submicron SnO_2_ particles. d) NH_3_ yields and FEs of flower‐like β‐Bi_2_O_3_ at different potentials. e) NH_3_ yields and f) FEs of SnO_2_ particles. a,b,d) Reproduced with permission.^[^ ^71^ ^]^ Copyright 2019, Wiley‐VCH. c,e,f) Reproduced with permission.^[^ ^72^ ^]^ Copyright 2018, Royal Society of Chemistry.

Apart from transition metals, Bi and Sn in the main group behave as NRR electrocatalysts with weak hydrogen adsorption. Bi nanosheets and Bi nanodendrites are active for the NRR due to the suppression of the HER and the morphology advantage.^[^ ^69^, ^70^ ^]^ Chang et al. reported that flower‐like β‐Bi_2_O_3_ (**Figure** **6**a,b) with a big surface area was an efficient electrocatalyst in neutral solution, of which the optimal NH_3_ yield and FE attained were 19.92 μg h^−1^ ${\text{mg}}_{\text{cat}\text{.}}^{-1}$ and 4.30%, respectively (Figure 6d).^[^ ^71^ ^]^ Cubic submicron SnO_2_ particles (Figure 6c) on carbon cloth (SnO_2_/CC) with a brilliant NRR property were fabricated via a one‐step hydrothermal method. Figure 6e,f shows that such SnO_2_ attained an NH_3_ yield of 1.47 × 10^−10^ mol s^−1^ cm^−2^ and FE of 2.17%, severally.^[^ ^72^ ^]^

### 3.2 Oxygen Vacancy

One of the more attractive advantages of using NPMOs as catalytic materials lies in their OVs. Such point defects can serve as chemisorption and activation sites for gas molecules such as N_2_. The electronic properties of NPMOs can be significantly changed by rational engineering of OVs in their lattices and surfaces, achieving a more efficient heterogeneous catalysis process.^[^ ^73^ ^]^ The OV introduction improves their conductivity and structural stability and further affects their performance to a certain extent. OVs can first efficaciously capture the metastable electrons and then deliver them into the antibond of N_2_ to recognize the electron receptor–donor process.^[^ ^74^ ^]^ OVs have been universally verified to be effective in activating catalysts, especially on the surfaces of metal oxides.

**Figure 7:**
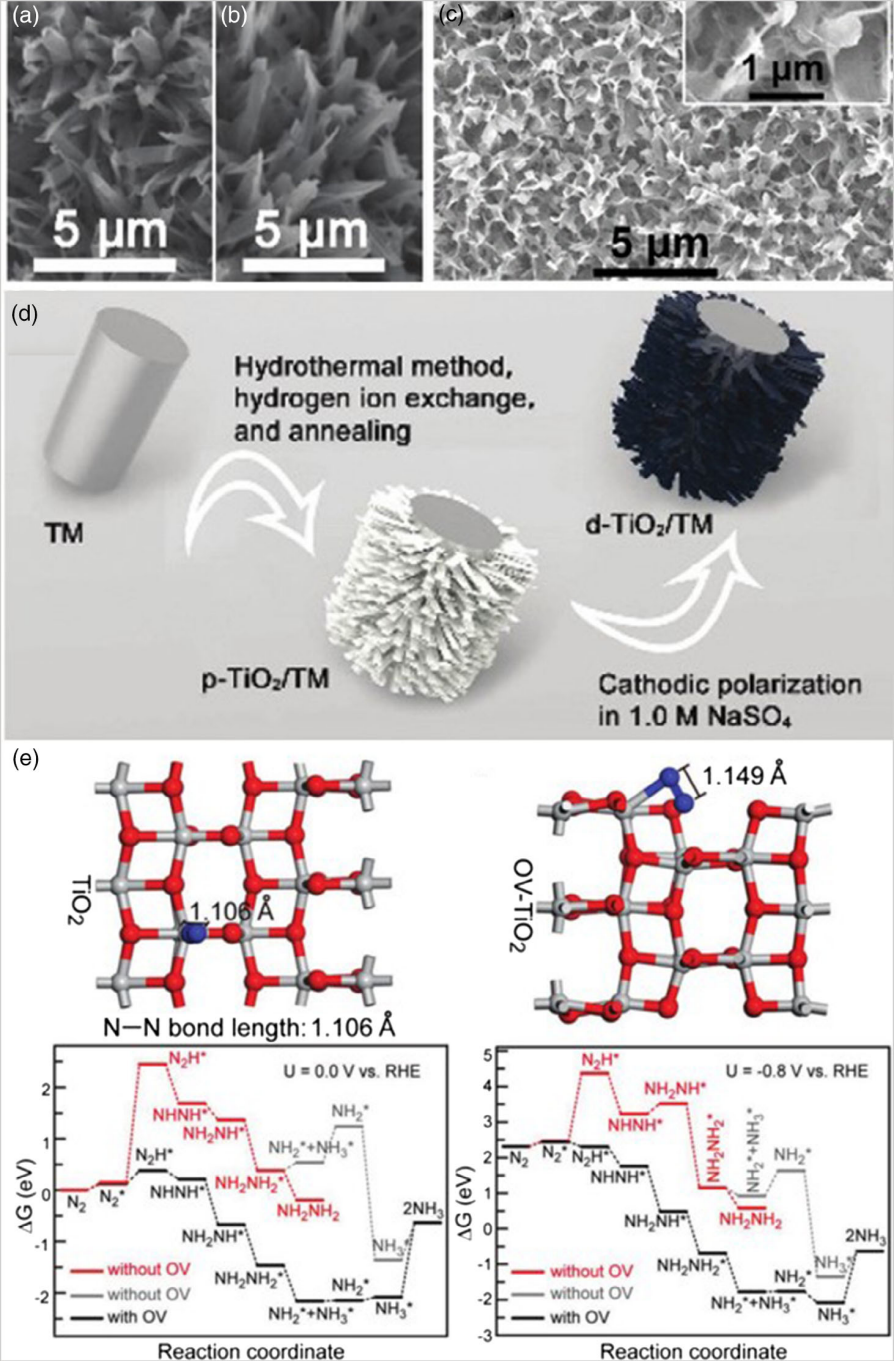
SEM images for a) d‐TiO_2_/TM, b) p‐TiO_2_/TM, and c) TiO_2_/Ti nanosheets. d) Process of fabrication of d‐TiO_2_/TM. e) Adsorption configurations of N_2_ molecule on perfect TiO_2_ surface and at the OV site of OV‐TiO_2_ and free energy profile of NRR process on anatase (010) surface with and without OV at potential of 0.0 V and at potential of −0.8 V. a,b,d) Reproduced with permission.^[^ ^49^ ^]^ Copyright 2019, Royal Society of Chemistry. c,e) Reproduced with permission.^[^ ^76^ ^]^ Copyright 2018, American Chemical Society.

Because it is a promising NRR catalyst, some researchers paid attention to designing TiO_2_ by cathodic electrochemical polarization and thermal treatment in an inert gas environment to introduce OVs. Defective TiO_2_ on a Ti mesh (d‐TiO_2_/TM) (**Figure** **7**a) was prepared by cathodic electrochemical polarization of p‐TiO_2_/TM (Figure 7b). Figure 7d gives the fabrication process of p‐TiO_2_/TM and d‐TiO_2_/TM. The NH_3_ yield increased from 0.17 × 10^−10^ to 1.24 × 10^−10^ mol s^−1^ cm^−2^ with an FE boosted from 0.95% to 9.17%.^[^ ^49^ ^]^ Fang et al. reported OV‐contained TiO_2_ (OV‐TiO_2_) nanosheets by a thermal treatment under a H_2_/Ar atmosphere and reached an NH_3_ yield of 35.6 μg h^−1^ ${\text{mg}}_{\text{cat}\text{.}}^{-1}$ and FE of 5.3%.^[^ ^75^ ^]^ Figure 7e shows that DFT calculations were conducted on the (010) surface of TiO_2_ with and without OVs. It also shows the free energy distribution in the process of the NRR on the (101) surface at 0 and −0.8 V versus a reversible hydrogen electrode (RHE). The DFT calculations demonstrated that the existence of OVs in TiO_2_ greatly reduced the energy barrier for the NRR. After 12 times electrolysis cycling tests, NH_3_ yields and FEs almost have no changes and TEM image still remains good. Another report about a TiO_2_ nanosheet array on a Ti plate (TiO_2_/Ti) (Figure 7c) with an NH_3_ yield of 9.16 × 10^−11^ mol s^−1^ cm^−2^ and FE of 2.5% demonstrated that OVs in situ magnify the adsorption and activation of N_2_.^[^ ^76^ ^]^ OV formation not only enhances the adsorption and activation of N_2_ but exposes more active sites in defective TiO_2_.

Fu et al. reported that Ta_2_O_5_ nanorods with rich OVs achieved an NH_3_ yield of 15.9 μg h^−1^ ${\text{mg}}_{\text{cat}\text{.}}^{-1}$ and an FE of 8.9%.^[^ ^77^ ^]^ The vapor hydrolysis–synthesized Ta_2_O_5_ nanorods further generated OVs in the process of hydrolysis by ethylene glycol at a high temperature. DFT calculations disclosed that N_2_ can be absorbed at the OV sites due to the coordination with two Ta atoms contiguous to the OVs. It is easier for the step of N_2_ adsorption on the surface of defective Ta_2_O_5_ than defect‐free Ta_2_O_5_ in the NRR process.

Zhang et al. designed OV‐MoO_2_ nanosheets using a modified chemical vapor deposition (CVD) process at an atomic level, achieving an NH_3_ yield of 12.20 μg h^−1^ ${\text{mg}}_{\text{cat}\text{.}}^{-1}$ and FE of 8.2%.^[^ ^78^ ^]^ DFT calculations disclosed that OVs in MoO_2_ layers were beneficial for the proton transfer step via selective stabilization of N_2_H* and destabilizing N_2_H_2_* via a distal or alternating hybrid path, thereby lowering the activation energy barrier from 1.49 to 0.36 eV with respect to the OV‐free one. DFT calculations suggest that inert N≡N triple bond could be activated by a single tungsten atom anchored on N‐doped graphyne. Inspired by this, Kong et al. demonstrated that 2D WO_3_ nanosheets OVs‐richness (OVs‐WO_3_) achieve an NH_3_ yield of 17.28 μg h^−1^ ${\text{mg}}_{\text{cat}\text{.}}^{-1}$ and a FE of 3.0%, outstripping the defect‐free WO_3_ nanosheets (6.47 μg h^−1^ ${\text{mg}}_{\text{cat}\text{.}}^{-1}$ and 1.02%).^[^ ^79^ ^]^

MnO_2_ with a OV nanowires array on a Ti mesh (OV‐MnO_2_) obtained an NH_3_ yield of 1.63 × 10^−10^ mol s^−1^ cm^−2^ and a FE of 11.4%, superior to its pristine MnO_2_ counterpart (2.3 × 10^−10^ mol s^−1^ cm^−2^ and 1.96%).^[^ ^80^ ^]^ DFT calculations manifested that the N_2_ adsorption enhanced on the OV‐MnO_2_ surface because of the stronger electronic interaction between the Mn_6*c*_ atoms and N_2_. Due to the favorable modulation of the electronic structure, Ni‐based materials have attracted considerable attention. Li et al. used the plasma technique, with its characteristics of being versatile, rapid, and energy‐saving, to engineer NiO nanosheets with enriched OVs, exhibiting an NH_3_ yield of 29.1 μg h^−1^ ${\text{mg}}_{\text{cat}\text{.}}^{-1}$ and FE of 10.8%.^[^ ^81^ ^]^

Because of the adaptable transition between Ce^4+^ and Ce^3+^, CeO_2_ has brilliant electronic or ionic conductivity and is considered an excellent material for investigating defect‐rich catalysts.^[^ ^82^ ^]^ Xu et al. utilized hydrogen‐reduced CeO_2_ nanorods, achieving an NH_3_ yield of 16.4 μg h^−1^ ${\text{mg}}_{\text{cat}\text{.}}^{-1}$ and FE of 3.7%, which is 2.8 times higher than that of the CeO_2_ nanorod precursor.^[^ ^83^ ^]^

According to these specific research works, we can summarize that OVs were generated by thermal treatment in an inert atmosphere, chemical reduction, and ion doping. We choose the most appropriate method to design catalysts based on their characteristic properties.

### 3.3 Heteroatom Doping

Heteroatom doping has been widely regarded as another valid approach to form coordination with the environment and modulate the electronic structure, accelerating the mobility of charge carriers and creating more active sites to boost the electrocatalytic activity.^[^ ^84^ ^]^ Heteroatom doping can be divided into metal element doping and nonmetal element doping.

#### 3.3.1 Metal Atom Doping

Metal doping is an effective pathway to design the compound material, enhance conductivity, and optimize absorption of the intermediates. DFT calculations manifest that metal sites with low chemical valence have the potential to enhance the electron‐donating ability to the π* antibonding orbitals of the N_2_ molecule.^[^ ^85^ ^]^ Consequently, the electrocatalytic activity will increase to a certain extent.

**Figure 8:**
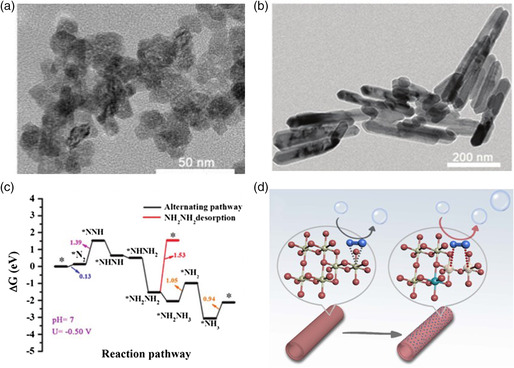
TEM images of a) Fe–TiO_2_ nanoparticles and b) V–TiO_2_ nanorods. c) Free energy diagram of the HER on pure and V–TiO_2_ (110) surface at *U* = −0.50 V and free energy diagram of the NRR on the V_2‐2_ site at *U* = −0.50 V. d) Schematic illustration of N_2_ fixation and activation. a,c) Reproduced with permission.^[^ ^86^ ^]^ Copyright 2019, Wiley‐VCH. b) Reproduced with permission.^[^ ^87^ ^]^ Copyright 2019, Wiley‐VCH. d) Reproduced under the terms of the CC‐BY 4.0 license.^[^ ^88^ ^]^ Copyright 2019, The Authors, published by Springer Nature.

The electrocatalytic performance of TiO_2_ can be improved by metal atom doping, including Fe, V, Cu, Mn, and Zr. Previously, we explained that Fe and V were crucial for biological N_2_ fixation and VO_2_,^[^ ^56^ ^]^ Fe_3_O_4_,^[^ ^67^ ^]^ and Fe_2_O_3_^[^ ^68^ ^]^ were reported as electrocatalysts for the NRR. Wu et al. reported Fe‐doped TiO_2_ nanoparticles (**Figure** **8**a) and V‐doped TiO_2_ nanorods (Figure 8b), achieving an NH_3_ yield of 25.47 and 17.73 μg h^−1^ ${\text{mg}}_{\text{cat}\text{.}}^{-1}$ and FE of 25.6% and 15.3%, respectively.^[^ ^86^, ^87^ ^]^ DFT calculation results revealed that the doping of Fe into TiO_2_ (101) increased the number of OVs. But the enhancement by V doping is caused by the synergistic effect of Ti and V. Figure 8c shows the free energy of the NRR process on the TiO_2_ (110) surface at *U* = −0.50 V. In Wu et al.'s work, the FE is higher than that of other research results and the electrolyte is LiClO_4_ because of which alkali metal ions suppress the HER to some extent. In addition, Cao et al. reported Zr^4+^ doping of anatase TiO_2_, achieving an NH_3_ yield of 8.90 μg h^−1^ ${\text{mg}}_{\text{cat}\text{.}}^{-1}$ and FE of 17.3%.^[^ ^88^ ^]^ The schematic illustration of N_2_ fixation is shown in Figure 8d. It suggests that Zr doping generates more OVs and bi‐Ti^3+^, which has a lower activation barrier as the most effective catalytic center of chemisorption and polarization N_2_. Ti^3+^ plays a significant role in TiO_2_ but what kinds of Ti^3+^ defect states improve the NRR activity in TiO_2_ is not clear. Cu ions with mixed valences of Cu^1+^ and Cu^2+^ can spontaneously modulate the OV concentration, which can induce different electronic defect states in transition‐metal oxides.^[^ ^89^ ^]^ Inspired by this, Wu et al. demonstrated that mixed‐valent Cu‐doped TiO_2_ behaved as an efficacious dopant to attune the OV concentration and Ti^3+^ formation, which is beneficial for generating different Ti^3+^ 3*d* ^1^ defect states localized below the Fermi energy.^[^ ^90^ ^]^ The synergistic effects of Cu^1+^–Ti^4+^, Ti^3+^–Ti^4+^, and Ti^3+^–Ti^3+^ all contribute to the enhanced NRR performance. Similarly, the typical dopant element Mn has the same effect on TiO_2_ by generating bi‐Ti^3+^. Zhang and co‐workers uncovered that Ti_4*c*_^3+^ was the active site for N_2_ binding and activation.^[^ ^91^ ^]^

Apart from defect‐rich CeO_2_, researchers also designed CeO_2_ by metal atom doping to improve the NRR activity. Theoretical calculation studies exposed that single atomic Cu substitution on the CeO_2_ (110) surface could augment to three OVs around each Cu site. Zhang et al. designed Cu‐doped CeO_2_ nanorods (Cu‐CeO_2_) attaining an NH_3_ yield of 5.3 × 10^−10^ mol s^−1^ cm^−2^ and FE of 19.1%.^[^ ^92^ ^]^ It was the Cu doping that effectively facilitated the concentration of multiple OVs in CeO_2_ and significantly boosted the NRR activity. In CeO_2_, Cu doping was apt to replacing Ce^3+^ sites by Cu^2+^. This rendered a decrease of Ce^3+^ ratio in the Ce^4+^, resulting in a decrease of oxygen vacancies around Ce^3+^ sites. But the increasing Cu content led to great increase of oxygen vacancies around Cu^2+^ sites, which were the real active centers. Xie et al. reported Cr‐doped CeO_2_ nanorods achieving an NH_3_ yield of 16.82 μg h^−1^ ${\text{mg}}_{\text{cat}\text{.}}^{-1}$ and an FE of 3.84%.^[^ ^93^ ^]^ It was a Cr atom that increased the OVs in CeO_2_. But different from Cu–CeO_2_, there was an increase of Ce^3+^ sites due to the Cr^3+^ reducibility, meaning a great increase of OVs around Ce^3+^ sites. In addition, Chu et al. reported that Fe doping could cause a morphology change form crystalline CeO_2_ nanoparticles to partially amorphous Fe–CeO_2_ nanosheets, filling with OVs.^[^ ^94^ ^]^ The NH_3_ yield and the FE achieved were 26.2 μg h^−1^ ${\text{mg}}_{\text{cat}\text{.}}^{-1}$ and 14.7%, respectively.

In addition, a commercial indium‐tin oxide glass (ITO/G) has been reported as a NRR electrocatalyst.^[^ ^95^ ^]^ Due to the In doping, the conductivity of SnO_2_ increases, leading to enhanced charge transfer to improve the NRR performance.

#### 3.3.2 Nonmetal Atom Doping

Apart from metal atom doping, nonmetal atom doping has become a research concentration as well. The most generally used nonmetal dopants are B, F, and N. By introducing different negative‐charge dopants and adjusting the electron acceptor–donor behavior of NPMOs, the charge transfer of NPMOs can be improved and the adsorption energy of reaction intermediates can be optimized, which favor the catalytic activity and reaction kinetics.

**Figure 9:**
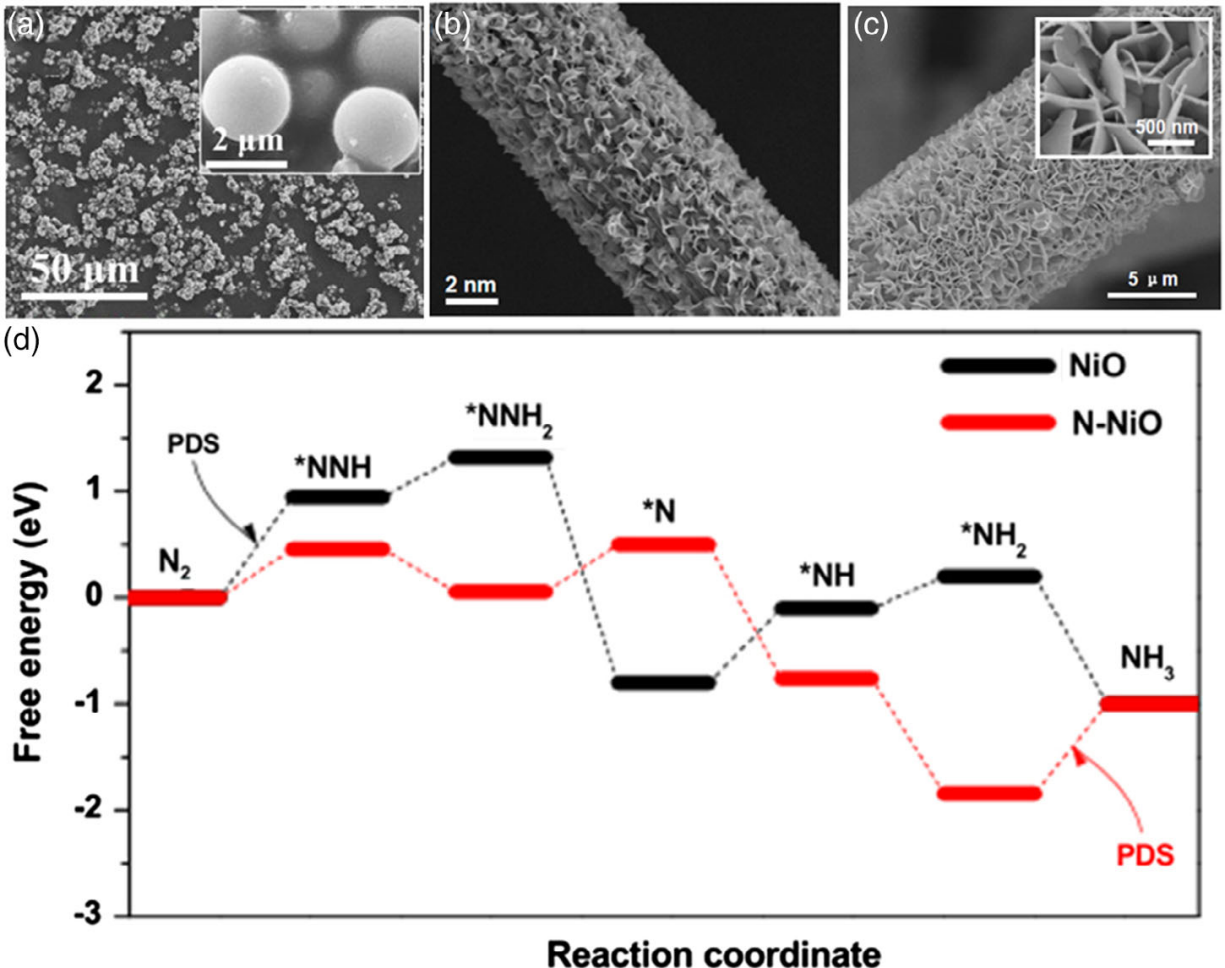
SEM images of a) B‐TiO_2_ microparticles, b) F‐SnO_2_ nanosheets, and c) N‐NiO nanosheets array. d) Adsorption of *NNH on N‐NiO and free energy diagrams of distal NRR pathway on NiO and N‐NiO at zero applied energy. a) Reproduced with permission.^[^ ^97^ ^]^ Copyright 2018, American Chemical Society. b) Reproduced with permission.^[^ ^99^ ^]^ Copyright 2019, American Chemical Society. c,d) Reproduced with permission.^[^ ^100^ ^]^ Copyright 2019, Wiley‐VCH.

Zheng and co‐workers reported that B is an important doping element that induces electron deficiency, which is beneficial for the NRR performance.^[^ ^96^ ^]^ Wang et al. reported B‐doped TiO_2_ microparticles (**Figure** **9**a) with the interstitial B serving as a three‐electron donor, and the NH_3_ yield and FE attained were 14.4 μg h^−1^ ${\text{mg}}_{\text{cat}\text{.}}^{-1}$ and 3.4%, respectively.^[^ ^97^ ^]^ Chu et al. reported the structural engineering of MnO_2_ nanosheets by B doping, which was found to effectively enrich OVs on the MnO_2_ nanosheets.^[^ ^98^ ^]^ It was B that induced lattice distortion predominantly and removed adjacent lattice oxygen to generate OVs. Such an electrocatalyst reached an NH_3_ yield of 54.2 μg h^−1^ ${\text{mg}}_{\text{cat}\text{.}}^{-1}$ with an FE of 16.8%.

SnO_2_ has a wider bandgap at the Fermi level and is typical semiconductor. Due to its dissatisfied conductivity, Chu and co‐workers prepared F‐doped SnO_2_ (F‐SnO_2_) mesoporous nanosheets (Figure 9b) grown on carbon cloth to reinforce its conductivity, achieving an NH_3_ yield of 19.3 μg h^−1^ ${\text{mg}}_{\text{cat}\text{.}}^{-1}$ and FE of 8.3%.^[^ ^99^ ^]^ After F doping, there was a negative shift of the conduction band of SnO_2_ toward the Fermi level, meaning metallic characteristics of F‐SnO_2_.

The N element has a higher electronegativity, which is favorable for the adsorption of N_2_. Chu and co‐workers also synthesized a N‐doped NiO (N‐NiO) nanosheets array (Figure 9c) on carbon cloth, achieving an NH_3_ yield of 22.7 μg h^−1^ ${\text{mg}}_{\text{cat}\text{.}}^{-1}$ and FE of 7.3%.^[^ ^100^ ^]^ DFT calculations disclosed that the N doping enhanced the surface conductivity, increased the *d*‐band center, and promoted *NNH stabilization. Figure 9d shows that N‐NiO changed the potential‐determining step (*N_2_ → *NNH) of NiO from the first hydrogenation step to the last NH_3_ release step (*NH_2_ → NH_3_).

### 3.4 Carbon Hybridization

In general, the unsatisfactory conductivity of NPMOs greatly confines their potentially brilliant NRR performance. Combining catalysts with conductive substrates is a forceful method to solve this problem. Synergetic effects are commonly induced by hybridization, which lead to generate more active sites, advanced charge transfer, and ameliorated intermediate adsorption.

The low electronic conductivity of V_2_O_3_ handicaps its application in the field of electrocatalysis. MOFs are known as appropriate self‐templates for metal oxide/carbon with conductivity compound materials.^[^ ^101^ ^]^ Therefore, Zhang et al. prepared shuttle V_2_O_3_/C by Ar annealing of a solvothermally synthesized V MOF precursor, with the NH_3_ yield and FE of 12.3 μg h^−1^ ${\text{mg}}_{\text{cat}\text{.}}^{-1}$ and 7.28%, respectively.^[^ ^102^ ^]^ Compared with V_2_O_3_, the NH_3_ yield increased from 2.0. to 12.3 μg h^−1^ ${\text{mg}}_{\text{cat}\text{.}}^{-1}$ due to the faster charge transfer.

Reduced graphene oxide (rGO) is a brilliant 2D conductive substrate due to its high conductivity, chemical stability, and high surface area.^[^ ^103^, ^104^ ^]^ Chu and co‐workers did a series of studies on metal oxides supported on rGO or graphene, including CuO,^[^ ^105^ ^]^ SnO_2_,^[^ ^106^ ^]^ MoO_2_,^[^ ^107^ ^]^ NiO,^[^ ^108^ ^]^ CoO,^[^ ^109^ ^]^ and ZnO.^[^ ^110^ ^]^ According to the DFT calculations, SnO_2_/defect graphene exhibited a smaller work function of 4.831 eV than SnO_2_ (6.128 eV), which demonstrated that SnO_2_/defect graphene had a higher Fermi level energy and could stimulate the donation of more valence electrons to the absorbed N_2_. Consequently, the strongly electronically coupled SnO_2_/defect graphene brought about enhanced conductivity and decreased work function, which was expected to enhance the NRR activity by accelerating the reaction kinetics and promoting the N_2_ adsorption and activation.^[^ ^106^ ^]^ TiO_2_‐supported by rGO (TiO_2_–rGO) showed higher conductivity and Zhang et al. reported that TiO_2_–rGO was active for NRR, reaching an NH_3_ yield of 15.13 μg h^−1^ ${\text{mg}}_{\text{cat}\text{.}}^{-1}$ and FE of 3.3%.^[^ ^111^ ^]^ Due to the wide bandgap of Cr_2_O_3_, it was an efficacious strategy to hybridize it with rGO, which enhanced the electron transfer.^[^ ^112^ ^]^

## 4 Recent Advances in NPMO Photocatalysts for the NRR

NPMOs are widely studied in heterogeneous photocatalysts because of their favorable electronic structure, excited lifetimes, and light adsorption properties. Many efforts have been dedicated to optimize the dynamic behavior of photogenerated carriers, such as introducing vacancies, regulating the nanostructure, and doping heteroatoms. In this section, we discuss the advances of TiO_2_‐based materials and other NPMO‐based materials for photocatalysis N_2_ reduction.

### 4.1 TiO_2_‐Based Materials

TiO_2_ has been regarded as a candidate photocatalyst due to its light absorption, charge transport, and surface adsorption,^[^ ^113^, ^114^ ^]^ and research of TiO_2_‐based materials for photocatalysis NRR is flourishing.

In 1977, Schrauzer and Guth first reported photocatalytic N_2_ to NH_3_ by TiO_2_.^[^ ^36^ ^]^ Later, there has been more and more research on TiO_2_ toward the photocatalytic NRR. In 1988, Bourgeois et al. reported that pristine TiO_2_ displayed photocatalytic N_2_ fixation performance by air annealing. The thermal pretreatment generated surface defects, introducing a defect or impurity state into the semiconductor bandgap.^[^ ^115^ ^]^ Later, Xie et al. probed the precise NRR process on the TiO_2_ (100) surface.^[^ ^116^ ^]^ The initial N_2_ adsorption, the activation of the N≡N, and the N—N cleavage were all efficiently promoted by TiO_2_ photogenerated electrons, surface hydroxylation, and their synergistic effects.

**Figure 10:**
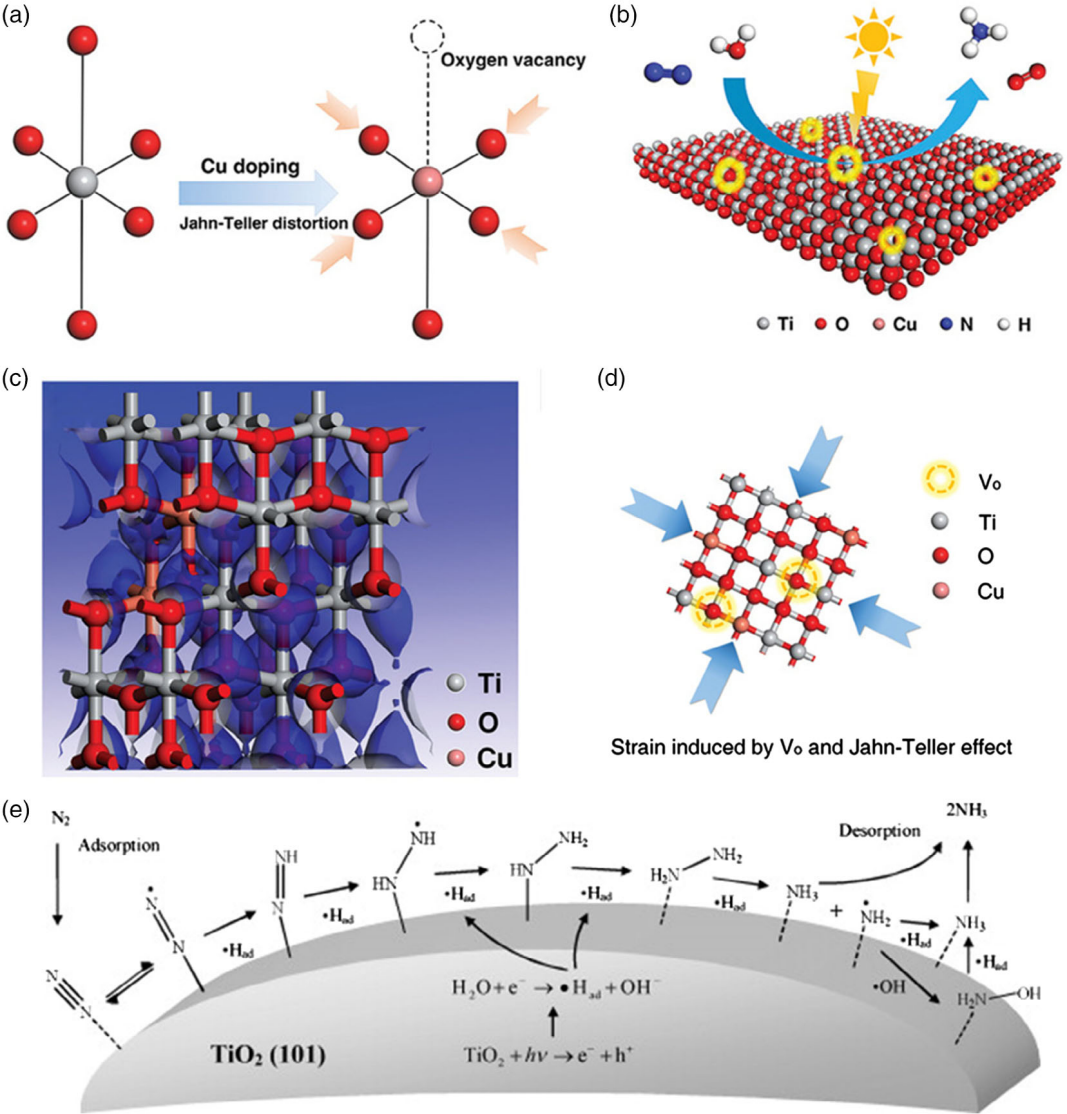
a) Polyhedral representation of a TiO_6_ octahedron and a defective octahedron with OVs resulting from Cu doping at a Ti site. b) Photocatalytic N_2_ fixation process on the surface of ultrathin TiO_2_ nanosheets with OVs and engineered strain. c) Electron density diagram for TiO_2_–OV–strain. d) 2D structural model of TiO_2_ nanosheets with OVs and engineered strain. e) N_2_ reduction scheme on the surface of TiO_2_ (101). a‐d) Reproduced with permission.^[^ ^119^ ^]^ Copyright 2019, Wiley‐VCH. e) Reproduced with permission.^[^ ^121^ ^]^ Copyright 2014, Elsevier.

Similar to electrocatalysts, introducing OVs is favorable for enhancing the photocatalysis performance.^[^ ^117^ ^]^ Hirakawa et al. reported that a commercial TiO_2_ with many surface OVs successfully produced NH_3_ by irradiating UV light and inletting N_2_.^[^ ^118^ ^]^ Interestingly, OVs can produce substantial strain in photocatalysts. Recently, it was demonstrated that ultrathin CuCr layered double hydroxide (LDHs) nanosheets containing abundant OVs improved the N_2_ adsorption properties and photoinduced charge transport. Zhao et al. reported OV‐TiO_2_ nanosheets containing 6% Cu (**Figure** **10**a) leading to improving the N_2_ photofixation performance.^[^ ^119^ ^]^ Figure 10b shows the photocatalytic NRR process on the surface of TiO_2_ nanosheets with OVs, which together with Jahn–Teller distortions of Cu^+^ centers resulted in compressive strain, which stabilized the OVs in the photocatalysts. The introduction of OVs and a strain effect contributed to the electron density around Ti or O atoms becoming larger (Figure 10c,d). Therefore, the OVs and strain in transition metal oxides enhance catalytic activity synergistically. Gong and co‐workers reported a plasmon‐enhanced amorphous TiO_2_ photoelectrode by atomic layer deposition (ALD) with surface OVs, offering higher carrier concentration and more active cites.^[^ ^120^ ^]^ Due to the ALD, OVs could be confined at the very surface region of TiO_2_ without affecting the bulk properties, which not only facilitated surface reaction between excited electrons and adsorbed N_2_ but also avoided recombination from undesired bulk defects.

With appropriate heteroatom doping, including Fe, Mg, Ce, and V, there are more defects in TiO_2_, resulting in modified adsorption properties.^[^ ^120^ ^]^ Metal ion doping also contributes to the absorption spectrum shifting to the visible region.^[^ ^120^ ^]^ Zhao et al. prepared Fe‐TiO_2_ nanoparticles by a two‐step hydrothermal method, contributing to a 3.84 times higher quantum yield than pristine TiO_2_.^[^ ^121^ ^]^ The dopant of Fe^3+^ on the TiO_2_ surface boosted the trapping of e^−^ and h^+^ by forming Fe^2+^ and Fe^4+^ to impede charge recombination and holes. The unstable Fe^2+^ and Fe^4+^ transferred e^−^ and h^+^ to Ti^4+^ and •OH^−^, generating Ti^3+^ and •OH, respectively. The surfacial Ti^3+^ provided an amount of active sites for N_2_ fixation by behaving as an electron donor, leading to dissociation of the triple bond. The N_2_ reduction scheme is shown in Figure 10e.

Ileperuma et al. studied Mg‐doped TiO_2_ with enhanced photocatalytic activity compared with pristine TiO_2_.^[^ ^122^ ^]^ In their research, the optimum doping level is 2–4%. By doping with Mg, there is a narrower depletion layer width in TiO_2_, affecting the rate of band bending. This could facilitate the electron tunneling to proceed more easily at the interphase, consequently enhancing electron transfer. Later, they performed research about Ce‐ and V‐doped TiO_2_.^[^ ^123^ ^]^ The V‐doped TiO_2_ catalyst shows n‐type semiconductor behavior at pH=3, whereas the Ce‐doped TiO_2_ possesses p‐type behavior at pH=12.5.

### 4.2 Other NPMO‐Based Materials

**Figure 11:**
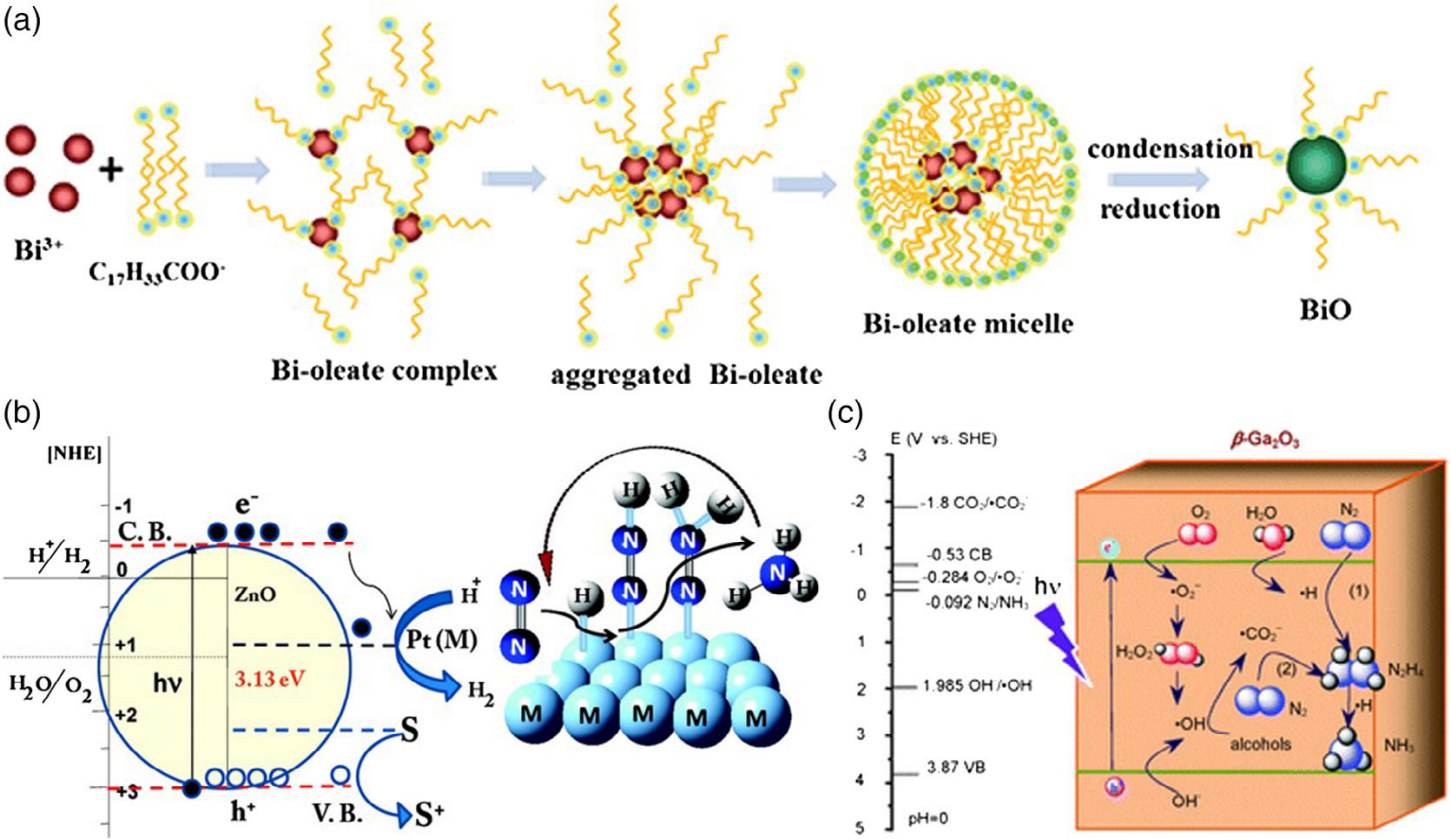
a) Schematic illustration of the whole synthesis procedure for the BiO quantum dots. b) Schematic representation of concurrent hydrogen production and nitrogen reduction. c) Speculated direct and indirect electron transfer pathways. a) Reproduced with permission.^[^ ^126^ ^]^ Copyright 2017, Royal Society of Chemistry. b) Reproduced with permission.^[^ ^127^ ^]^ Copyright 2010, American Chemical Society. c) Reproduced with permission.^[^ ^128^ ^]^ Copyright 2015, Royal Society of Chemistry.

Recently, there have been various catalytic applications of Bi‐based materials.^[^ ^124^, ^125^ ^]^ Sun et al. focused on low‐valent Bi (II) and demonstrated that BiO without additional reducing agents is an ideal model for the NRR due to its empty 6*d* orbitals and high electron donating power for N_2_ adsorption and activation.^[^ ^126^ ^]^ As shown in **Figure** **11**a, N_2_ was activated by supplying electrons to the 6*d* orbital of Bi and accepted the lone pair electrons from three Bi atoms to its antibonding orbit.

ZnO is another excellent semiconductor material optics due to its wide bandgap. Janet et al. used the wet chemical etching method to prepare ZnO and the N_2_ photofixation yielded around 86 μmol h^−1^ $g_{\text{cat}\text{.}}^{-1}$.^[^ ^127^ ^]^ A schematic shows the whole process of water splitting at the semiconductor metal interface along with the N_2_ activation through the insertion of N_2_ to the Pt—H bond (Figure 11b).

Zhao et al. reported that mesoporous β‐Ga_2_O_3_ nanorods, a wide‐bandgap semiconductor, were used in N_2_ photoreduction.^[^ ^128^ ^]^ The photoactivity of β‐Ga_2_O_3_ was operated in the presence of different alcohols, including tert‐butanol (TBA), methanol, and ethanol, which formed •${\text{CO}}_{2}^{-}$ in situ. The performance of N_2_ to NH_3_ was enhanced by reducing ability of •${\text{CO}}_{2}^{-}$ dramatically. In addition, the presence of O_2_ in the N_2_ accelerated the formation of •${\text{CO}}_{2}^{-}$, which boosted the reducing energy (Figure 11c).

As an n‐type semiconductor, WO_3_ is also a promising photocatalyst because of its brilliant electron and proton conductivity. Li et al. reported WO_3_ with carbon decoration as a photocatalyst with an NH_3_ yield of 205 μmol h^−1^ $g_{\text{cat}\text{.}}^{-1}$.^[^ ^129^ ^]^ Pothole‐rich WO_3_ nanosheets are also a photocatalyst for direct nitrate synthesis.^[^ ^130^ ^]^ Mo is a typical doping element for the NRR. In past research, SrMoO_4_ and BiMoO_6_ were reported as photocatalysts for the NRR.^[^ ^131^, ^132^ ^]^ Moreover, Xiong's group reported Mo‐W_18_O_49_ nanowires and revealed the doping of Mo species contributed to multisynergetic effects on N_2_ activation and dissociation.^[^ ^133^ ^]^ The active sites of Mo–W not only polarized the adsorbed N_2_, but also enhanced metal–oxygen covalency in the lattice. Mo doping facilitated the photocatalytic NRR by raising the defect‐band center to preserve the energy of photoexcited electrons.

Fe is universally in the nitrogenase and Haber–Bosch process ascribed to its good interaction with dinitrogen. Both Fe_2_O_3_ and Fe_3_O_4_ have N_2_ photocatalytic activity. Khader et al. synthesized α‐Fe_2_O_3_ that was effective in photoactivating N_2_.^[^ ^134^ ^]^ Recently, Lashgariand and Zeinalkhani reported that the 1:1 ratio nanocomposite of Fe_2_O_3_–TiO_2_ and Pd‐loaded Fe_2_O_3_–TiO_2_ were active for photofixation.^[^ ^135^ ^]^ Thus, as Fe_2_O_3_ was incorporated into TiO_2_, due to the extended light absorption of visible light in Fe_2_O_3_, the coupled heterojunction nanocomposite materials played a key role in the N_2_ photoreduction process.

In addition, hydrous oxides Cu_2_O•*x*H_2_O, WO_3_•H_2_O, and Sm_2_O_3_•*x*H_2_O/V_2_O_3_•*x*H_2_O were also studied as photocatalysts in the NRR.^[^ ^136^, ^137^, ^138^ ^]^

## 5 Conclusion

Highly efficient NH_3_ production at ambient conditions is an urgent research duty for the continuous development of contemporary society. Low‐voltage electricity and solar energy can act as alternative energy sources for NH_3_ production, whereas the Haber–Bosch process exhausts massive energy and releases greenhouse gases. Electrocatalytic and photocatalytic artificial N_2_ fixation have fascinated rising research attention due to the following virtues: 1) The reactive materials (N_2_ and H_2_O) are infinite and the reactive conditions are moderate, leading to lower cost and 2) electricity and solar are clean and renewable sources; therefore, the artificial NRR process alleviates environmental issues with no carbon emission. The reported studies and the performance of electrocatalysts and photocatalysts are summarized in **Table** **1** and **2**. According to these reports, NPMO catalysts show good stability, which refers to the change of catalysts’ activity and selectivity with time. Most of the catalysts display chemical and structural stability. The NH_3_ yields and FEs almost have little change after long tests and repeated experiments. In addition, the components and morphologies remain greatly after cycle tests. Nevertheless, the current researches are still superficial and defective. The NRR still faces grand challenges. In our Review, we describe the recent progress in NPMO electrocatalysts and photocatalysts for the NRR and highlight the approaches to overcome the poor NRR activity of pristine NPMO by heteroatom doping to engineer its surface‐active sites and introduction of OVs. Eventually, a brief summary of and future perspective on this research field are also proposed. 1) It is worth engineering catalysts rationally to overcome their lower intrinsic catalytic reactivity and selectivity for the NRR. Herein, previously, we talked about the pristine NPMO catalysts first. Then, we narrated OV introduction and heteroatom doping, which both enrich the active sites and enhance the catalytic performance apparently.

**Table 1:** Comparison of NRR performance of NPMO electrocatalysts

Catalyst	Electrolyte	NH_3_ yield (μg h^−1^ ${\text{mg}}_{\text{cat}}^{-1}$)	FE	Potential (V) versus RHE	Ref.
Y_2_O_3_	0.1 m Na_2_SO_4_	1.06 × 10^−10^ mol s^−1^ cm^−2^	2.53%	−0.90V	[46]
p‐TiO_2_	0.1 m HCl	0.17 × 10^−10^ mol s^−1^ cm^−2^	0.95%	−0.15V	[49]
d‐TiO_2_	0.1 m HCl	1.24 × 10^−10^ mol s^−1^ cm^−2^	9.17%	−0.90V	[49]
OVs‐TiO_2_	0.005 m H_2_SO_4_	35.6	5.30%	−0.80V	[75]
Ti/TiO_2_	0.1 m Na_2_SO_4_	9.16 × 10^−10^ mol s^−1^ cm^−2^	2.50%	−0.70V	[76]
Fe‐TiO_2_	0.5 m LiClO_4_	25.47	25.60%	−0.40V	[86]
V‐TiO_2_	0.5 m LiClO_4_	17.73	15.30%	−0.40V	[87]
Cu‐TiO_2_	0.5 m LiClO_4_	21.31	21.99	−0.55	[90]
Zr‐TiO_2_	0.1 m KOH	8.90	17.30%	−0.45V	[88]
Mn‐TiO_2_	0.1 m Na_2_SO_4_	20.05	11.93%	−0.50V	[91]
B‐TiO_2_	0.1 m Na_2_SO_4_	14.4	3.40%	−0.80V	[96]
TiO_2_/RGO	0.1 m Na_2_SO_4_	15.13	5.30%	−0.80V	[111]
CuO/RGO	0.1 m Na_2_SO_4_	1.8 × 10^−10^ mol s^−1^ cm^−2^	3.90%	−0.75V	[105]
SnO_2_/RGO	0.1 m Na_2_SO_4_	25.6	7.10%	−0.50V	[106]
MoO_2_/RGO	0.1 m Na_2_SO_4_	37.4	6.60%	−0.35V	[107]
NiO/RGO	0.1 m Na_2_SO_4_	18.6	7.80%	−0. 70V	[108]
CoO/RGO	0.1 m Na_2_SO_4_	21.5	8.30%	−0.60V	[109]
ZnO/RGO	0.1 m Na_2_SO_4_	17.7	6.40%	−0.65V	[110]
ZrO_2_	0.1 m HCl	24.74	5.0%	−0.45V	[50]
NbO_2_	0.05 m H_2_SO_4_	11.6	32.0%	−0.65V	[51]
Nb_2_O_5_	0.1 m HCl	43.6	9.26%	−0.55V	[52]
Nb_2_O_5_	0.1 m Na_2_SO_4_	1.58 × 10^−10^ mol s^−1^ cm^−2^	2.26%	−0.60V	[53]
VO_2_	0.1 m Na_2_SO_4_	14.85	3.97%	−0.70V	[56]
V_2_O_3_/C	0.1 m Na_2_SO_4_	12.3	7.28%	−0.60V	[102]
Cr_2_O_3_	0.1 m HCl	28.13	8.56%	−0.90V	[57]
Hollow Cr_2_O_3_	0.1 m Na_2_SO_4_	25.3	6.78%	−0.90V	[58]
Ta_2_O_5_	0.1 m HCl	15.9	8.9%	−0.60V	[77]
MoO_3_	0.1 m HCl	4.80 × 10^−10^ mol s^−1^ cm^−2^	1.9%	−0.40V	[62]
MoO_2_	0.1 m HCl	12.2	8.20%	−0.15V	[78]
WO_3_	0.1 m HCl	17.28	7.00%	−0.30V	[79]
MnO	0.1 m Na_2_SO_4_	1.11 × 10^−10^ mol s^−1^ cm^−2^	8.02%	−0.39V	[66]
MnO_2_	0.1 m Na_2_SO_4_	2.3 × 10^−11^ mol s^−1^ cm^−2^	1.96%	−0.50V	[80]
B‐MnO_2_	0.5 m LiClO_4_	54.2	16.80%	−0.20V	[98]
Mn_3_O_4_	0.1 m Na_2_SO_4_	11.6	3.00%	−0.80V	[65]
Fe_3_O_4_	0.1 m Na_2_SO_4_	5.6 × 10^−11^ mol s^−1^ cm^−2^	2.6%	−0.40V	[67]
Fe_2_O_3_	0.1 m Na_2_SO_4_	15.9	0.94%	−0.80V	[68]
NiO	0.1 m Na_2_SO_4_	29.1	10.8%	−0.50V	[81]
N‐NiO	0.5 m LiClO4	22.7	7.30%	−0.50V	[100]
CeO_2_	0.1 m Na_2_SO_4_	16.4	3.70%	−0.40V	[83]
Cu/CeO_2_	0.1 m Na_2_SO_4_	5.3 × 10^−10^ mol s^−1^ cm^−2^	19.1%	−0.45V	[92]
Cr/CeO_2_	0.1 m Na_2_SO_4_	16.82	3.84%	−0.70V	[93]
Fe/CeO_2_	0.5 m LiClO_4_	26.2	14.70%	−0.50V	[94]
Bi_2_O_3_	0.1 m Na_2_SO_4_	19.92	4.30%	−0.80V	[71]
SnO_2_	0.1 m Na_2_SO_4_	1.47 × 10^−10^ mol s^−1^ cm^−2^	2.17%	−0.70V	[72]
ITO/G	0.5 m LiClO_4_	1.06 × 10^−10^ mol s^−1^ cm^−2^	6.17%	−0.40V	[95]
F/SnO_2_	0.1 m Na_2_SO_4_	19.3	8.60%	−0.45V	[99]

**Table 2:** Comparison of NRR performance of NPMO photocatalysts

Material	Light source	Scavenger	NH_3_ yield	Ref.
OVs‐TiO_2_	>280 nm	2‐Propanol	35.0 μmol h^−1^ g^−1^_cat._	[115]
OVs‐TiO_2_	200‐800 nm	/	78.9 μmol h^−1^ g^−1^_cat._	[116]
Fe‐TiO_2_	=254 nm	Ethanol	400 μmol h^−1^ g^−1^_cat._	[121]
Mg‐TiO_2_	UV light	/	10.3 μmol h^−1^ g^−1^_cat._	[122]
V‐TiO_2_	UV light	/	28 μmol h^−1^ g^−1^_cat._	[123]
Ce‐TiO_2_	UV light	/	30 μmol h^−1^ g^−1^_cat._	[123]
BiO	Full spectra	Methanol	1226 μmol h^−1^ g^−1^_cat._	[126]
ZnO	UV light	EDTA‐2Na	35 μmol h^−1^ g^−1^_cat._	[127]
β‐Ga_2_O_3_	*λ* = 254 nm	TBA	/	[128]
WO_3_	Full spectra	/	205 μmol h^−1^ g^−1^_cat._	[129]
Mo‐W_18_O_49_	Full spectra	Na_2_SO_3_	195.5 μmol h^−1^ g^−1^_cat._	[133]
Fe_2_O_3_	UV light	/	100 μmol h^−1^ m^−2^	[134]
Cu_2_O•*x*H_2_O/CuCl	UV light	/	70 mM h^−1c^ ^*f*^	[136]
Sm_2_O_3_/V_2_O_3_	UV light	/	44 μmol h^−1c^ ^*f*^	[138]

OVs in NPMOs play a critical role in boosting the catalysis performance. Strategies to create such OVs differ but generally lack fine control over OVs’ concentration and location (bulk or surface).^[^ ^139^ ^]^ In addition, surface OVs in NPMOs suffer from oxidation, thus leading to activity decay. Practical strategies for stabilizing surface OVs are therefore expected. Qiu et al. designed p–n heterojunction CeO_2_/Co_3_O_4_ and OVs were generated by the coupled heterojunction interface, which rendered rapid interfacial charge transfer from CeO_2_ to Co_3_O_4_.^[^ ^140^ ^]^ Note that characterization to verify and elucidate OVs such as electron paramagnetic resonance (EPR), X‐ray absorption fine structure spectroscopy (XAFS), and scanning tunneling microscopy (STM) should be performed. DFT is also a powerful method that constructs theoretical models at the atomic and molecular level to analyze changes in electronic structure and assess the catalytic mechanism of materials bearing OVs. For photocatalysts, DFT calculations could help estimate bandgap changes, charge separation, as well as adsorption and activation of the substances involved in catalytic reactions. The energy barrier for the rate‐limiting step of a catalytic reaction can also be calculated to evaluate how OVs impact the efficiency and selectivity of the catalysis. Therefore, there will be a promising future for metal oxides with OVs by DFT calculation forecasting, regulating rationally, and verifying precisely.

The role of dopants is to change the NPMO's surface chemical bonds to render oxygen atoms close to the dopants. Therefore, the active centers may be the oxygen atoms near the dopant or the dopant itself. The main synthesis methods for doped oxides include solid‐state synthesis, coprecipitation, sol–gel synthesis, and electrochemical synthesis. However, no matter which method is used, we could not know if the doped oxides and the dopants are homogeneous or even make sure whether the dopants are small oxide clusters on the surface or not. Therefore, advanced and precise characterization methods using photons, electrons, or chemical behaviors are needed for further demonstration. Generally, XRD is not a precise method because the doped oxide has the same structure as the pristine oxide. Moreover, it is clear whether the subtle lattice parameter shift is to be ascribed to the dopant or the small size of crystallites. Extended X‐ray absorption fine structure (EXAFS) could locate the dopant in the solid exactly. Another efficacious technique is X‐ray photoelectron spectroscopy (XPS), which presents the surface concentration of the dopant and oxidation states of atoms on the surface. Electron microscopy, including SEM and electron stimulated X‐ray emission spectroscopy (EDX), provides morphological information. In addition, spectroscopy, including UV optical spectroscopy (UV–vis), Raman spectroscopy, and Fourier transformed infrared spectroscopy (FTIR) also could identify the presence of dopants.^[^ ^89^ ^]^ 2) Amorphous metal oxides (AMOs) will offer us a new direction in the field of NRR as most of the catalysts we discussed before are crystals and the research of AMOs in the NRR is limited. AMOs with highly disordered 3D arrangement present high catalytic performance in energy electrocatalysis.^[^ ^141^ ^]^ Rich defects in AMOs also bestow higher catalytic site densities on them.^[^ ^142^ ^]^ Due to their strong flexibility, AMO electrocatalysts can self‐regulate themselves under working conditions and offer both volume and surface sites, enabling more facilitated electrolyte diffusion.^[^ ^143^ ^]^ In contrast, crystalline materials only provide surface catalysis. With these merits of amorphous materials, further and deep research in NRR fields are needed to take more efforts. For photocatalysts, amorphous structures have a smaller bandgap and more defects, which enhance visible light absorption and promote the migration and separation of photogenerated charge carries.^[^ ^144^ ^]^ 3) Stability is an essential criterion of whether a catalyst can be used in practice. In fact, metal oxides dissolve more or less in acidic media. Although NPMOs show good stability for acidic NRR in previous reports, these research works^[^ ^51^, ^75^ ^]^ lack reliable characterizations (such as in situ electrochemical quartz crystal microbalance and inductivity coupled plasma) to describe the leaching rate of oxide ions. Indeed, the stability of NPMOs can be demonstrated by cycling/long‐term stability tests. But for accurate quantification of this critical criterion, we need more details concerning NPMOs’ mass change during electrocatalysis. Meanwhile, the duration of stability testing in the NRR should be extended. Apart from these, strategies such as engineering special heterojunctions to improve NPMOs’ stability also count. For instance, the stability of Pt nanoparticles can be much enhanced by constructing a Pt–ITO–graphene triple junction.^[^ ^145^ ^]^ Likewise, preparing NPMOs on suitable substrates may also stabilize them for N_2_ reduction. 4) Theoretical calculations and computational methods have made it feasible to investigate catalysts at the atomic level. Furthermore, there are many factors remaining unclear because the NRR is a greatly complex multistep reaction. The DFT method has been widely used as a practical tool to predict and forecast the possible active sites, adsorption energies, catalytic pathways, intermediates, and rate‐determining step of the reaction. It is a powerful and effective tool to combine experimental results and theoretical analysis, which has contributed to disclosing the molecular‐scale reaction mechanisms of the NRR. Thus, more focus should be paid to establish sound models closer to factual reaction systems. 5) NO_*x*_ contaminants and other N‐containing substances exist in the process of the NRR. To prevent misjudgment of the tested catalyst, accurate detection of the source of the produced ${\text{NH}}_{4}^{+}$ based on an isotopic labeling test via ${\text{NH}}_{4}^{+}$‐selective electrodes, ion chromatography, and/or a quantitative NMR method is a necessity. Moreover, eliminating pollution from nitrogen sources before the NRR process is also important.

## Conflict of Interest

The authors declare no conflict of interest.
